# Frequent and Persistent Salivary Gland Ectasia and Oral Disease After COVID-19

**DOI:** 10.1177/0022034521997112

**Published:** 2021-03-03

**Authors:** E.F. Gherlone, E. Polizzi, G. Tetè, R. De Lorenzo, C. Magnaghi, P. Rovere Querini, F. Ciceri

**Affiliations:** 1 Vita-Salute San Raffaele University, Milan, Italy; 2 Department of Dentistry, IRCCS Ospedale San Raffaele, Milan, Italy; 3 Division of Immunology, Transplantation and Infectious Disease, IRCCS Ospedale San Raffaele, Milan, Italy; 4 Division of Hematology, IRCCS Ospedale San Raffaele, Milan, Italy

**Keywords:** epidemiology, hospital dentistry, infectious disease(s), oral pathology, salivary diagnostic, SARS-CoV-2

## Abstract

The clinical picture of coronavirus disease 2019 (COVID-19) in various target organs has been extensively studied and described. However, relatively little is known about the characteristics of oral cavity involvement. This is surprising, considering that oral mucosal and salivary gland cells are known targets for the direct replication of severe acute respiratory syndrome coronavirus 2 (SARS-CoV-2) and that the presence of the virus in saliva is a source of transmission of the infection. The aim of our study was to investigate the presence and prevalence of oral manifestations in COVID-19 survivors. We profiled the oral involvement in 122 COVID-19 survivors that were hospitalized and followed up at a single-referral university hospital in Milan, Italy, between July 23, 2020 and September 7, 2020, after a median (interquartile range) time from hospital discharge of 104 (95 to 132) d. We found that oral manifestations, specifically salivary gland ectasia, were unexpectedly common, with oral manifestations being detected in 83.9% while salivary gland ectasia in 43% of COVID-19 survivors. Salivary gland ectasia reflected the hyperinflammatory response to SARS-CoV-2, as demonstrated by the significant relationship with C-reactive protein (CRP) and lactate dehydrogenase (LDH) levels at hospital admission, and with the use of antibiotics during acute disease. Both LDH levels and antibiotic administration survived as independent predictors of salivary gland ectasia at multivariable analysis. Temporomandibular joint abnormalities, facial pain, and masticatory muscle weakness were also common. Overall, this retrospective and prospective cohort study of COVID-19 survivors revealed that residual damage of the oral cavity persists in the vast majority of patients far beyond clinical recovery, and suggests that the oral cavity represents a preferential target for SARS-CoV-2 infection. Further studies are needed to clarify the connection between SARS-CoV-2 infection and oral disorders.

*“No man is an island, entire of itself; every man is a piece of the continent, a part of the main.” (John Donne)*

## Introduction

Viral diseases often affect the oral cavity. For example, human immunodeficiency virus (HIV) infection may initially present with oral lesions, human papillomavirus (HPV) infection may increase the risk of developing oral squamous cell carcinoma, and oral involvement in hepatitis B virus (HBV) and hepatitis C virus (HCV) infections has been documented (Jiang and Dong 2017; Parisi et al. 2017; Ottria et al. 2018; Nayyar et al. 2020). Less characterized is the impact of acute, rather than chronic, viral infections on the oral cavity. We have scarce evidence of oral manifestations associated with severe acute respiratory syndrome (SARS) or Middle East respiratory syndrome (MERS) (De Wit et al. 2016; Otter et al. 2016). Knowledge on oral involvement in coronavirus disease 2019 (COVID-19) is also limited. This is surprising since salivary glands and epithelial cells of the oral mucosa are known to express angiotensin-converting enzyme 2 (ACE2), the best characterized entry receptor for SARS-CoV-2 into cells (Pascolo et al. 2020). Moreover, the virus itself has been detected in the saliva of most COVID-19 patients (Xu et al. 2020). Therefore, the oral cavity seems to be a privileged and accessible environment for the interaction of SARS-CoV-2 with target cells and with the mucosal immune system (Dos Santos et al. 2020).

The dental team can thus contribute to improving the quality of prevention and treatment of COVID-19 and to increasing the scientific knowledge regarding this relatively new disease, despite the limitations caused by the production of aerosols during the use of rotating instruments, and by the required short distance between operators and the patient’s mouth (Peng et al. 2020). In addition to the direct effects of the virus on the oral cavity, the anti-viral inflammatory response may worsen underlying oral disorders, specifically those related to autoimmunity and immune deficiencies (Dziedzic and Wojtyczka 2020; Yuen et al. 2020).

Drugs may also play their part. High doses of corticosteroids could precipitate fungal infections such as oral candidiasis, while antiviral drugs may cause stomatitis, aphthous ulcers, and dry mouth in a consistent fraction of patients (Scully and Dios 2001). Most patients are given antibiotics effective against a wide range of gram-positive and gram-negative bacteria, impacting on the oral microbiome and on the homeostasis of the oral cavity (Jensen et al. 2015).

Patients with COVID-19 often undergo intubation, assisted external ventilation, and tracheostomy (Zangrillo et al. 2020). These procedures cause hyposalivation, which exacerbates various pre-existing injuries of the oral cavity and can result in bacterial aspiration pneumonia (Wu et al. 2020). SARS-CoV-2 appears to have a tropism for nerves, and damage to sensory neurons has been hypothesized to be involved in the frequent occurrence of anosmia and ageusia (Vaira et al. 2020). Neuronal injury may also affect facial muscle tone and impair the secretory function of salivary glands.

COVID-19 leaves behind substantial clinical sequelae (De Lorenzo et al. 2020). The present study investigates whether oral manifestations may occur as part of COVID-19, and whether they persist after viral clearance and clinical recovery.

## Methods

### Study Population and Design

This is a retrospective and prospective cohort study that follows STROBE (Strengthening the Reporting of Observational Studies in Epidemiology) guidelines, and is included in a large single-center observational study, the COVID-BioB study, conducted at San Raffaele University Hospital in Milan. All patients aged ≥18 y, admitted to the Emergency Department of San Raffaele University Hospital from February 25, 2020, for COVID-19 were consecutively enrolled in the COVID-BioB study (Rovere-Querini et al. 2020). COVID-19 diagnosis was based on a positive SARS-CoV-2 nasopharyngeal swab on real-time reverse transcription-polymerase chain reaction (RT-PCR) in the presence of clinical and/or radiological signs of COVID-19. Patients discharged after viral clearance (De Lorenzo et al. 2020; Farina et al. 2020), defined as 2 negative consecutive swabs, were subsequently evaluated at the COVID-19 Follow-up Outpatient Clinic of San Raffaele University Hospital. Consecutive patients evaluated at follow-up from July 23, 2020 to September 7, 2020 were included in the present study. The COVID-BioB study protocol, compliant with the Helsinki declaration, was approved by the San Raffaele Hospital Ethics Committee (CE-OSR, protocol no.34/int/2020) and registered on ClinicalTrials.gov (NCT04318366) (Rovere-Querini et al. 2020). All patients signed the informed consent.

### Follow-up Evaluation

As part of a multidisciplinary medical evaluation, all patients underwent extraoral and intraoral physical examination by an experienced dental specialist. Through the extraoral examination, we investigated the presence or absence of abnormalities in facial lymph nodes and in the temporomandibular joint (TMJ), and any facial asymmetries. With the intraoral examination, lips, cheeks, salivary glands, hard palate, oropharynx, tongue, mucous membranes, and frenula were assessed (Saita et al. 2013). A case-record form, specifically developed for the present study, was used to collect detailed data on family and past medical history of the oral cavity. The form was completed during the follow-up evaluation by the dental specialist through direct patient interview (Appendix). Data regarding the acute phase of COVID-19 were collected through a retrospective scrutiny of the medical records in the presence of the patient at the follow-up visit.

The following variables were included in the medical assessment: age, sex, comorbidities (presence of arterial hypertension [HTN], coronary artery disease [CAD], diabetes mellitus [DM], chronic kidney disease [CKD], active neoplasia, chronic obstructive pulmonary disease [COPD]), family history of periodontal disease, smoking habit, time since last dental visit, COVID-19 history (transfer to intensive care unit [ICU], administration of noninvasive ventilation [NIV], degree of respiratory failure quantified as the ratio of partial oxygen pressure [PaO_2_] to inspired oxygen fraction [FiO_2_], PaO_2_/FiO_2_), C-reactive protein (CRP) and lactate dehydrogenase (LDH) serum levels, absolute lymphocyte count at hospital admission, therapy received, and involvement of the oral cavity and nearby structures that arose during or after COVID-19 (TMJ abnormalities, facial pain due to facial muscle weakness, oral ulcers, dry mouth, facial tingling, trigeminal neuralgia, altered taste and/or smell, white hairy tongue, facial asymmetry, latero-cervical, retro-cervical, and submandibular lymphadenopathy, anomalies of lips, cheeks, salivary glands, hard palate, oropharynx, mucous membranes, and frenula). Abnormalities of the TMJ were assessed by the presence of joint clicks and/or pain when placing 2 fingers at the level of the mandibular condyles and inviting the patient to open and close the mouth. Facial pain due to reduced tone of facial muscles and dry mouth were reported by the patient when completing the case-record form. Reduced tone of facial muscles was assessed by palpation. The co-presence of facial pain and decreased facial muscle tone defined causality. Salivary glands were defined as being ectasic when they appeared swollen, with a patent duct, and no pus leaking. Abnormalities of the gums and soft tissues were also considered.

### Statistical Analysis

Categorical variables were expressed as count (percentage [%]) and continuous variables as median (interquartile range [IQR]). Chi-square test or Fisher test were employed to compare categorical variables between groups. Differences in continuous variables between groups were assessed using Mann-Whitney U test. Univariable and multivariable logistic regression analyses were performed to investigate the contribution of individual variables to the development of salivary gland ectasia and dry mouth. All variables that emerged as predictors (*P* < 0.05) at univariable analysis were used as covariates in the multivariate model. Missing data were not imputed. All statistical analyses were performed using R statistical package (version 4.0.0, R Foundation for Statistical Computing), with a 2-sided significance level set at *P* < 0.05.

## Results

A total of 122 patients admitted to San Raffaele University Hospital for COVID-19 were included. Of these patients, 75% were male, and the median (IQR) age was 62.5 (53.9 to 74.1) y. Main comorbidities, transfer to ICU, NIV administration, disease severity at onset, and therapy during hospital stay are reported in Tables 1 and 2. Median (IQR) time from discharge to follow-up visit was 104 (95 to 132.8) d. Follow-up evaluation comprised assessment of TMJ abnormalities, masticatory muscle weakness, oral ulcers, dry mouth, facial tingling, trigeminal neuralgia, altered taste and/or smell, facial asymmetry, latero-cervical, retro-cervical and submandibular lymph-adenopathy, anomalies of lips, tongue, cheeks, salivary glands, hard palate, oropharynx, mucous membranes, and frenula. No patient reported oral cavity disorders prior to COVID-19, while at follow-up evaluation 101 (83.6%) COVID-19 survivors presented oral cavity or facial abnormalities (Fig.).

**Table 1.** Abnormalities in TMJ and Masticatory Muscle Weakness at Follow-up Visit in Patients with COVID-19 (*n* = 122).

		Abnormalities in TMJ			Masticatory Muscle Weakness		
	Overall (*n* = 122)	Absent (*n* = 113)	Present (*n* = 9)	*P* Value	Absent (*n* = 100)	Present (*n* = 22)	*P* Value
Age, y	62.5 (53.9-74.1)	63.2 (55-75)	48.8 (46.1-62.2)	0.027	63.7 (55.3-76.3)	57 (47.8-66.3)	0.06
Female sex	30 (24.6)	26 (23)	4 (44.4)	0.30	26 (26.3)	4 (17.4)	0.53
Comorbidities							
HTN	50 (41)	48 (42.5)	2 (22.2)	0.40	44 (44.4)	6 (26.1)	0.17
CAD	12 (9.8)	12 (10.6)	0 (0)	0.65	9 (9.1)	3 (13)	0.85
DM	17 (13.9)	15 (13.3)	2 (22.2)	0.81	15 (15.2)	2 (8.7)	0.64
CKD	9 (7.4)	9 (8)	0 (0)	0.83	9 (7.4)	2 (8.7)	1.00
Neoplasia	7 (5.7)	6 (5.3)	1 (11.1)	1.00	7 (7.1)	0 (0)	0.41
COPD	8 (6.6)	7 (6.2)	1 (11.1)	1.00	5 (5.1)	3 (13)	0.35
Smoking	48 (39.3)	46 (40.7)	2 (22.2)	0.46	39 (39.4)	9 (39.1)	1.00
Last odontoiatric visit							
<1 y prior to FU	68 (55.7)	62 (54.9)	6 (66.7)	0.56	54 (54.5)	14 (60.9)	0.84
≥1 y prior to FU	22 (18)	20 (17.7)	2 (22.2)		18 (18.2)	4 (17.4)	
≥3 y prior to FU	32 (26.2)	31 (27.4)	1 (11.1)		27 (27.3)	5 (21.7)	
Hospitalization for COVID-19	115 (94.3)	107 (94.7)	8 (88.9)	0.99	94 (94.9)	21 (91.3)	0.86
Transfer to ICU	30 (24.6)	28 (24.8)	2 (22.2)	1.00	26 (26.3)	4 (17.4)	0.53
Noninvasive ventilation	54 (44.3)	51 (45.1)	3 (33.3)	0.73	45 (45.5)	9 (39.1)	0.75
At hospital admission							
PaO_2_/FiO_2_	272.9 (185-323.8)	271.4 (166.3-319)	412.9 (353.2-476.4)	0.001	271.4 (193.4-319)	314.3 (138.3-338.1)	0.43
CRP (mg/dL)	81 (40.1-128.9)	82.8 (43.2-129.9)	48.9 (3.8-71.1)	0.09	87(42.1-139.4)	58.4 (33.2-81.5)	0.11
LDH (U/L)	351.5 (257.2-466)	357 (269.8-467.8)	229.5 (208-368.8)	0.07	358 (269-484)	328 (252-380)	0.24
Lymphocytes (×10^9^/L)	0.9 (0.6-1.3)	0.9 (0.6-1.2)	1.5 (0.6-1.7)	0.20	0.9 (0.6-1.2)	0.9 (0.7-1.3)	0.81
Therapy during hospital stay							
Steroids	36 (29.5)	35 (31)	1 (11.1)	0.37	31 (31.3)	5 (2.7)	0.50
Antibiotics	102 (83.6)	96 (85)	6 (66.7)	0.33	80 (80.8)	22 (97.5)	0.15
Biologics	38 (31.1)	35 (31)	3 (33.3)	1.00	34 (34.3)	4 (17.4)	0.18

Dichotomous variables were expressed as count (percentage), while continuous variables were expressed as median (interquartile range).

CAD, coronary artery disease; CKD, chronic kidney disease; COPD, chronic bronchopulmonary disease; COVID-19, coronavirus disease 2019; CRP, C-reactive protein; DM, diabetes mellitus; FU, follow-up; HTN, arterial hypertension; ICU, intensive care unit; LDH, lactate dehydrogenase; PaO_2_/FiO_2_, arterial oxygen partial pressure/fractional inspired oxygen; TMJ, temporomandibular joint.

**Table 2.** Salivary Gland Ectasia and Dry Mouth at Follow-up Visit in Patients with COVID-19 (*n* = 122).

	Salivary Glands Ectasia			Dry Mouth		
	Absent (*n* = 76)	Present (*n* = 46)	*P* Value	Absent (*n* = 92)	Present (*n* = 30)	*P* Value
Age, y	61.7 (50.6-74.4)	63.6 (58.7-72.9)	0.021	61.4 (51.6-70.8)	67.4 (57.4-77.4)	0.16
Female sex	23 (30.3)	7 (15.2)	0.09	24 (26.1)	6 (20)	0.67
Comorbidities						
HTN	33 (43.4)	17 (37)	0.60	38 (41.3)	12 (40)	1.00
CAD	5 (6.6)	7 (15.2)	0.21	8 (8.7)	4 (13.3)	0.70
DM	10 (13.2)	7 (15.2)	0.96	9 (9.8)	8 (26.7)	0.04
CKD	8 (10.5)	1 (2.2)	0.18	7 (7.6)	2 (6.7)	1.00
Neoplasia	5 (6.6)	2 (4.3)	0.91	5 (5.4)	2 (6.7)	0.99
COPD	5 (6.6)	3 (6.5)	1.00	2 (2.2)	6 (20)	0.002
Smoking	28 (36.8)	20 (43.5)	0.59	36 (39.1)	12 (40)	1.00
Last odontoiatric visit						
<1 y prior to FU	41 (53.9)	27 (58.7)	0.87	54 (58.7)	14 (46.7)	0.33
≥1 y prior to FU	14 (18.4)	8 (17.4)		14 (15.2)	8 (26.7)	
≥3 y prior to FU	21 (27.6)	11 (23.9)		24 (26.1)	8 (26.7)	
Hospitalization for COVID-19	69 (90.8)	46 (100)	0.086	85 (92.4)	30 (100)	0.27
Transfer to ICU	16 (21.1)	14 (30.4)	0.34	23 (25)	7 (23.3)	1.00
Noninvasive ventilation	29 (38.2)	25 (54.3)	0.12	41 (44.6)	13 (43.3)	0.99
At hospital admission						
PaO_2_/FiO_2_	284.8 (239.3-323.8)	266.7 (153.3-323.8)	0.21	272.1 (180.4-322.6)	276.2 (209.9-325)	0.80
CRP (mg/dL)	78.2 (26.8-114)	89.2 (49.8-178.8)	0.05	83.5 (44.1-128.9)	61 (40.1-117)	0.41
LDH (U/L)	315 (246.2-393)	424 (293-547.8)	0.01	358 (257.5-476)	329(252.5-408)	0.57
Lymphocytes (×10^9^/L)	1 (0.7-1.4)	0.8 (0.5-1)	0.04	0.9 (0.6-1.3)	0.8 (0.6-1.1)	0.68
Therapy during hospital stay						
Steroids	21 (27.6)	15 (32.6)	0.74	24 (26.1)	12 (40)	0.23
Antibiotics	59 (77.6)	43 (93.5)	0.04	77 (83.7)	25 (83.3)	0.99
Biologics	21 (27.6)	17 (37)	0.39	28 (30.4)	10 (33.3)	0.94

Dichotomous variables were expressed as count (percentage), while continuous variables were expressed as median (interquartile range).

CAD, coronary artery disease; CKD, chronic kidney disease; COPD, chronic bronchopulmonary disease; COVID-19, coronavirus disease 2019; CRP, C-reactive protein; DM, diabetes mellitus; FU, follow-up; HTN, arterial hypertension; ICU, intensive care unit; LDH, lactate dehydrogenase; PaO_2_/FiO_2_, arterial oxygen partial pressure/fractional inspired oxygen.

**Figure.**
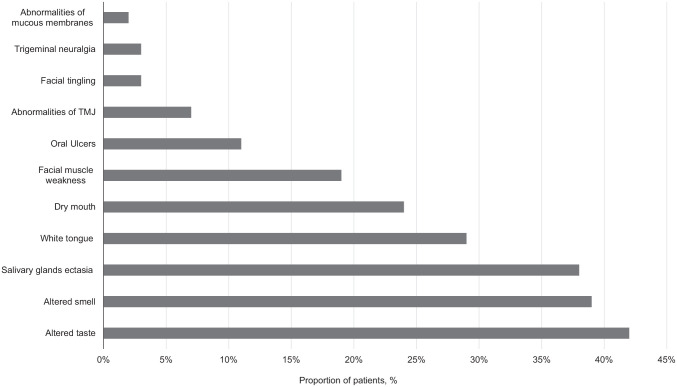
Oral cavity abnormalities lesions found in COVID-19 patients at the post-recovery follow-up. Lesions present in less than 3% of patients were not depicted. COVID-19, coronavirus disease 2019; TMJ, temporomandibular joint.

Salivary gland ectasia, dry mouth, TMJ abnormalities, and masticatory muscle weakness were frequent findings. Salivary gland ectasia was the most common oral manifestation, and was detected in 46 patients (38%), with a significant male preponderance. Patients who developed salivary gland ectasia had more severe COVID-19 and were significantly older (Table 2). Accordingly, patients with salivary gland ectasia had, upon hospital admission, higher levels of serum CRP and LDH, and lower absolute lymphocyte counts (Table 2). Moreover, the majority of patients (93%) with salivary gland ectasia received antibiotics during hospitalization. Univariable logistic regression analysis confirmed that the risk of developing salivary gland ectasia increases significantly with increasing CRP and LDH levels at hospital admission (Table 3). Antibiotic administration during COVID-19 also significantly increased the odds of developing salivary glands ectasia (Table 3). Notably, serum levels of LDH, which reflect overall necrosis, and antibiotic therapy survived as independent predictors of salivary gland ectasia at multivariable analysis (Table 3).

**Table 3.** Univariable and Multivariable Logistic Regression Analyses Predicting Salivary Glands Ectasia at Follow-up in Patients with COVID-19 (*n* = 122).

	Univariable Analysis			Multivariable Analysis		
	OR	95% CI	*P* Value	OR	95% CI	*P* Value
Age, y	1.019	0.99 to 1.048	0.18			
Female sex	0.41	0.15 to 1.018	0.006			
Smoking	1.32	0.62 to 2.78	0.46			
Comorbidities						
HTN	0.764	0.36 to 1.61	0.48			
CAD	2.55	0.76 to 9.11	0.48			
DM	1.18	0.40 to 3.34	0.75			
CKD	0.19	0.010 to 1.080	0.12			
COPD	0.99	0.19 to 4.24	0.99			
Hospitalization for COVID-19	1.043 × 10^7^	0.00 to NA	0.99			
Transfer to ICU	1.64	0.70 to 3.79	0.24			
Noninvasive ventilation	1.93	0.92 to 4.08	0.08			
At hospital admission						
PaO_2_/FiO_2_	0.99	0.99 to 1.001	0.33			
CRP (mg/dL)	1.005	1.00 to 1.009	0.02	0.99	0.99 to 1.00	0.22
LDH (U/L)	1.003	1.001 to 1.006	0.005	1.00	1.001 to 1.007	0.016
Lymphocytes (×10^9^/L)	0.715	0.37 to 1.04	0.23			
Therapy during hospital stay						
Steroids	1.24	0.55 to 2.75	0.59			
Antibiotics	4.13	1.28 to 18.48	0.03	8.34	1.47 to 158.19	0.049
Biologics	1.53	0.69 to 3.36	0.28			

CAD, coronary artery disease; CKD, chronic kidney disease; COPD, chronic bronchopulmonary disease; COVID-19, coronavirus disease 2019; CRP, C-reactive protein; DM, diabetes mellitus; HTN, arterial hypertension; ICU, intensive care unit; LDH, lactate dehydrogenase; OR, odds ratio; PaO_2_/FiO_2_, arterial oxygen partial pressure/fractional inspired oxygen.

Dry mouth was found at follow-up evaluation in 30% of patients. Thirteen patients had both dry mouth and salivary glands ectasia (Table 2). DM and COPD were found to be significantly associated with dry mouth (Table 4), and COPD survived as an independent predictor of dry mouth at multivariable analysis (Table 4).

**Table 4.** Univariable and Multivariable Logistic Regression Analyses Predicting Dry Mouth at Follow-up in Patients with COVID-19 (*n* = 122).

	Univariable Analysis			Multivariable Analysis		
	OR	95% CI	*P* Value	OR	95% CI	*P* Value
Age, y	1.02	0.99 to 1.05	0.19	1.004	0.97 to 1.040	0.81
Female sex	0.71	0.23 to 1.85	0.50			
Smoking	0.973	0.45 to 1.84	0.85			
Comorbidities						
HTN	0.947	0.40 to 2.18	0.89			
CAD	1.61	0.40 to 5.58	0.46			
DM	3.35	1.14 to 9.78	0.02	2.67	0.81 to 8.63	0.09
CKD	0.867	0.12 to 3.84	0.86			
COPD	1.12	2.42 to 80.20	0.004	9.10	1.80 to 68.49	0.01
Hospitalization for COVID-19	1.50 × 10^7^	0.00 to NA	0.99			
Transfer to ICU	0.913	0.32 to 2.32	0.85			
Noninvasive ventilation	1.93	0.92 to 4.08	0.08			
At hospital admission						
PaO_2_/FiO_2_	0.988	0.99 to 1.003	0.57			
CRP (mg/dL)	0.982	0.99 to 1.003	0.49			
LDH (U/L)	0.992	0.99 to 1.001	0.53			
Lymphocytes (×10^9^/L)	0.851	0.46 to 1.15	0.45			
Therapy during hospital stay						
Steroids	1.86	0.77 to 4.41	0.16			
Antibiotics	0.974	0.33 to 3.23	0.96			
Biologics	1.14	0.46 to 2.71	0.76			

Multivariable analysis was adjusted also for age, even though this variable did not reach statistical significance at univariable analysis.

CAD, coronary artery disease; CKD, chronic kidney disease; COPD, chronic bronchopulmonary disease; COVID-19, coronavirus disease 2019; CRP, C-reactive protein; DM, diabetes mellitus; HTN, arterial hypertension; ICU, intensive care unit; LDH, lactate dehydrogenase; NA, not measurable; OR, odds ratio; PaO_2_/FiO_2_, arterial oxygen partial pressure/fractional inspired oxygen.

TMJ abnormalities were found in 9 patients (7%). Patients with TMJ anomalies were significantly younger than patients without (Table 1). Masticatory muscle weakness was also a common event, found in 23 patients (19%) (Table 1). This feature might reflect different events, including direct and indirect nerve involvement. Dysgeusia and anosmia were detected in 14 (17%) and 12 (14%) patients, respectively. Four (3%) patients had facial tingling, 4 (2%) patients had trigeminal neuralgia, and a single patient (0.8%) had facial asymmetry. No association was detected between these features and patient or disease characteristics (not shown).

## Discussion

Evidence of oral cavity abnormalities in patients with COVID-19 is limited to a few case reports and case series (Dos Santos et al. 2020; Kitakawa et al. 2020). In the present study, we investigated the prevalence and type of oral manifestations of COVID-19 in a large and well-characterized cohort of survivors. We found that 83.6% of patients had anomalies of the oral cavity or nearby structures at approximately 3 mo after hospital discharge. The most common manifestations were salivary gland ectasia and dry mouth. A considerable proportion of patients had TMJ abnormalities and complained of facial pain associated with reduced facial muscle tone. No lesions occurred at the level of the lips and cheeks.

The high fraction of COVID-19 survivors that underwent oral sequelae after the infection, although striking, well agrees with current knowledge of SARS-CoV-2. The virus initially enters and replicates in the epithelial cells of the upper respiratory tract, as a consequence of the interaction of the S spike protein with ACE-2 receptors and subsequent cleavage by the transmembrane protease, serine 2 (TMPRSS2) enzyme. These events result in high local viral load and shedding, and significant transmissibility (Morris et al. 2020). ACE-2 and TMPRSS2 are both expressed in salivary glands and epithelial cells within the oral cavity (Brandão et al. 2020; Song et al. 2020; Usami et al. 2020), an observation that has raised the question of whether salivary glands might be productively infected during COVID-19 (Song et al. 2020). Although this contention needs formal proof, it would well fit with the almost universal involvement of salivary glands we observed. Moreover, SARS-CoV-2 is readily detectable in the saliva of patients with COVID-19, even if it remains controversial whether this reflects the clinical severity of the disease (Chen et al. 2020; Hanege et al. 2020; Sakanashi et al. 2020; To et al. 2020).

Patients in the present study were assessed well after effective viral clearance (Farina et al. 2020). Therefore, lingering involvement could only partially reflect the direct cytopathic action of the virus on infected cells of the oral tissues. It is more likely that oral involvement occurs as a consequence of the host inflammatory response, which is responsible for most morbidity and mortality in COVID-19. In agreement with a role of the early innate immune response, we found a strongly significant relationship between salivary gland ectasia and the levels of CRP, a marker of systemic inflammation, and LDH, a marker of overall necrosis, at the onset of clinical symptoms. LDH also survived as an independent predictor of salivary gland ectasia at multivariate analysis. LDH and CRP have been suggested as surrogates of COVID-19 severity (Zangrillo et al. 2020). Therefore, it is tempting to speculate that salivary gland abnormalities associate with a more severe course of COVID-19.

Of importance, anti-SARS-CoV-2 antibodies were found to be readily detectable in patients’ saliva for at least 3 mo after symptom onset (Isho et al. 2020; Pisanic et al. 2020), clearly pointing to the oral cavity as a privileged immune site during COVID-19.

To the best of our knowledge, this is the first systematic study of oral involvement in a large cohort of well-characterized COVID-19 survivors. However, oral involvement has been previously described. Three cases of herpetic-like orobuccal lesions were described in patients with SARS-CoV-2 infection, which improved within 3 to 10 d without any specific treatment (Martín Carreras-Presas et al. 2020). In our cohort, a single patient had a herpetic lesion (Guo et al. 2020). A patient presented a lesion of hemorrhagic origin, which was most likely traumatic, while angina-bullous hemorrhagic lesions of the oral mucosa and nonspecific stomatitis (Cruz Tapia et al. 2020) did not occur. Tongue abnormalities were limited to hairy white tongue (28%), a condition characterized by marked hypertrophy of the filiform papillae due to excess keratin production and/or poor oral hygiene (Stoopler et al. 2018; Tecco et al. 2018). In previous studies, length of hospital stay, NIV administration, orotracheal intubation, and long-term multi-drug therapy were associated with lesions in the oral cavity, loss of incisors due to intubation, and decreased saliva secretion (Wu et al. 2020). Accordingly, we observed that patients who received antibiotics during the acute phase of disease more commonly developed salivary gland ectasia, antibiotic therapy being an independent predictor of ectasia development. Antibiotic treatment was frequently used as both a preventive and therapeutic measure in patients with a more severe clinical picture during the first wave of the pandemic. Therefore, our observation may reflect a more severe course of COVID-19 in patients developing salivary gland ectasia. Also, oral microbiome derangement following antibiotics may predispose to development of gland lesions, although causality may be difficult to confirm. In contrast, steroid and anti-cytokine therapies were not associated with oral lesions.

Salivary gland ectasia was a common finding in our population, being present in more than one-third of patients. A previous study suggested a link between salivary gland ectasia and NIV administration (Bortolotti 2017). In our cohort, we failed to detect a connection between NIV use and salivary gland ectasia or dry mouth, implying that other mechanisms may be involved, possibly related to systemic inflammation, maladaptive immune responses, circulatory dysfunction, and neurological dysfunction (Yelins’ka et al. 2018; Aghagoli et al. 2020; Ciceri et al. 2020; Ghannam et al. 2020).

This study has limitations. First, we could not perform any functional or anatomical study of major salivary glands and could not explore the possible contribution of minor salivary glands to the clinical scenario. Nevertheless, our study was intended as an initial clinical assessment of the oral cavity. Certainly, an accurate clinical examination may not prescind from further instrumental exams to confirm clinical findings for an optimal patient management. Second, data on oral lesions prior to COVID-19 were collected retrospectively at the follow-up visit through patient interview, which may have led to estimation bias. Likewise, no information at admission and during hospital stay was available, as the unprecedented workload of healthcare professionals made oral cavity examination unfeasible in times of emergency. Moreover, at the time of the study a rapid test for the screening of SARS-CoV-2 in patient saliva was not available. Since SARS-CoV-2 has been demonstrated in a substantial fraction (3 out of 12) of convalescent patient saliva despite multiple negative nasopharyngeal swabs (Sakanashi et al. 2020), we cannot readily distinguish between features related to the potential local viral persistence and those related to the sequelae of the infection. Finally, the relationship between the use of antibiotics and salivary gland ectasia points to a potential role of the salivary microbiome, which we could not directly prove.

Altogether, our results clearly indicate that the oral cavity is a possible target of COVID-19, with alterations persisting in the vast majority of survivors well after clinical recovery. Further studies are needed to elucidate the connection between SARS-CoV-2 infection and oral disorders. The direct action of the virus, the effect of the inflammatory response on oral homeostasis, neurological mechanisms, and the impact of treatments, antibiotics in particular, should all be taken into account.

## Author Contributions

E.F. Gherlone, E. Polizzi, P. Rovere Querini, F. Ciceri, contributed to conception and design, critically revised the manuscript; G. Tetè, R. De Lorenzo, contributed to data acquisition, analysis, and interpretation, drafted the manuscript; C. Magnaghi, contributed to data acquisition, analysis, and interpretation, critically revised the manuscript. All authors gave final approval and agree to be accountable for all aspects of the work.

## Supplemental Material

sj-pdf-1-jdr-10.1177_0022034521997112 – Supplemental material for Frequent and Persistent Salivary Gland Ectasia and Oral Disease After COVID-19 Click here for additional data file. Supplemental material, sj-pdf-1-jdr-10.1177_0022034521997112 for Frequent and Persistent Salivary Gland Ectasia and Oral Disease After COVID-19 by E.F. Gherlone, E. Polizzi, G. Tetè, R. De Lorenzo, C. Magnaghi, P. Rovere Querini and F. Ciceri in Journal of Dental Research

## References

[bibr1-0022034521997112] Aghagoli G Marin BG Katchur NJ Chaves-Sell F Asaad WF Murphy SA. 2020. Neurological involvement in COVID-19 and potential mechanisms: a review. Neurocrit Care [epub ahead of print 13 July 2020]. doi:10.1007/s12028-020-01049-4 PMC7358290 32661794

[bibr2-0022034521997112] Bortolotti M. 2017. The cause of dry mouth during CPAP application. J Clin Sleep Med. 13(4):647. 28162149 10.5664/jcsm.6568 PMC5359345

[bibr3-0022034521997112] Brandão TB Gueiros LA Melo TS Prado-Ribeiro AC Nesrallah ACFA Prado GVB Santos-Silva AR Migliorati CA. 2020. Oral lesions in patients with SARS-CoV-2 infection: could the oral cavity be a target organ? Oral Surg Oral Med Oral Pathol Oral Radiol [epub ahead of print 18 Aug 2020]. doi:10.1016/j.oooo.2020.07.014 PMC7434495 32888876

[bibr4-0022034521997112] Chen L Zhao J Peng J Li X Deng X Geng Z Shen Z Guo F Zhang Q Jin Y, et al. 2020. Detection of SARS-CoV-2 in saliva and characterization of oral symptoms in COVID-19 patients. Cell Prolif. 53(12):e12923. 10.1111/cpr.12923 PMC7645955 33073910

[bibr5-0022034521997112] Ciceri F Beretta L Scandroglio AM Colombo S Landoni G Ruggeri A Peccatori J D’Angelo A De Cobelli F Rovere-Querini P, et al. 2020. Microvascular COVID-19 lung vessels obstructive thromboinflammatory syndrome (MicroCLOTS): an atypical acute respiratory distress syndrome working hypothesis. Crit Care Resusc. 22(2):95–97. 32294809 10.51893/2020.2.pov2 PMC10692450

[bibr6-0022034521997112] Cruz Tapia RO Peraza Labrador AJ Guimaraes DM Matos Valdez LH. 2020. Oral mucosal lesions in patients with SARS-CoV-2 infection. Report of four cases. Are they a true sign of COVID-19 disease? Spec Care Dentist. 40(6):555–560. 32882068 10.1111/scd.12520

[bibr7-0022034521997112] De Lorenzo R Conte C Lanzani C Benedetti F Roveri L Mazza MG Brioni E Giacalone G Canti V Sofia V, et al. 2020. Residual clinical damage after COVID-19: a retrospective and prospective observational cohort study. PLoS One. 15(10):e0239570. 10.1371/journal.pone.0239570 PMC7556454 33052920

[bibr8-0022034521997112] De Wit E van Doremalen N Falzarano D Munster VC. 2016. SARS and MERS: recent insights into emerging coronaviruses. Nat Rev Microbiol. 14(8):523–534. 27344959 10.1038/nrmicro.2016.81 PMC7097822

[bibr9-0022034521997112] Dos Santos JA Normando AGC da Silva RLC De Paula RM Cembranel AC Santos-Silva AR Guerra ENS. 2020. Oral mucosal lesions in a COVID-19 patient: new signs or secondary manifestations? Int J Infect Dis. 97:326–328. 32526392 10.1016/j.ijid.2020.06.012 PMC7280113

[bibr10-0022034521997112] Dziedzic A Wojtyczka R. 2020. The impact of coronavirus infectious disease 19 (COVID-19) on oral health. Oral Dis [epub ahead of print 18 Apr 2020]. doi:10.1111/odi.13359 PMC7264805 32304276

[bibr11-0022034521997112] Farina N Ramirez GA De Lorenzo R Di Filippo L Conte C Ciceri F Manfredi AA Rovere-Querini P. 2020. COVID-19: pharmacology and kinetics of viral clearance. Pharmacol Res. 161:105114. 10.1016/j.phrs.2020.105114 PMC7834389 32758635

[bibr12-0022034521997112] Ghannam M Alshaer Q Al-Chalabi M Zakarna L Robertson J Manousakis G. 2020. Neurological involvement of coronavirus disease 2019: a systematic review. J Neurol. 267(11):3135–3153. 32561990 10.1007/s00415-020-09990-2 PMC7304377

[bibr13-0022034521997112] Guo Y Yuan C Wei C. 2020. Emergency measures for acute oral mucosa diseases during the outbreak of COVID-19. Oral Dis [epub ahead of print 11 Apr 2020]. doi:10.1111/odi.13350 PMC7262343 32277533

[bibr14-0022034521997112] Hanege FM Kocoglu E Kalcioglu MT Celik S Cag Y Esen F Bayindir E Pence S Alp Mese E Agalar C. 2020. SARS-CoV-2 presence in the saliva, tears and cerumen of COVID-19 patients. Laryngoscope [epub ahead of print 23 Oct 2020]. doi:10.1002/lary.29218 33094833

[bibr15-0022034521997112] Isho B Abe KT Zuo M Jamal AJ Rathod B Wang JH Li Z Chao G Rojas OL Bang YM, et al. 2020. Persistence of serum and saliva antibody responses to SARS-CoV-2 spike antigens in COVID-19 patients. Sci Immunol. 5(52):eabe5511. 10.1126/sciimmunol.abe5511 PMC8050884 33033173

[bibr16-0022034521997112] Jensen SJ Hein L Lundgren B Bestle MH Mohr T Andersen MH Løken J Tousi H Søe-Jensen P Lauritsen AØ, et al.; Procalcitonin and Survival Study Group. 2015. Invasive candida infections and the harm from antibacterial drugs in critically ill patients: data from a randomized, controlled trial to determine the role of ciprofloxacin, piperacillin-tazobactam, meropenem, and cefuroxime. Crit Care Med. 43(3):594–602. 25493970 10.1097/CCM.0000000000000746

[bibr17-0022034521997112] Jiang S Dong Y. 2017. Human papilloma virus and oral squamous cell carcinoma: a review of HPV-positive oral squamous cell carcinoma and possible strategies for future. Curr Probl Cancer. 41(5):323–327. 28416242 10.1016/j.currproblcancer.2017.02.006

[bibr18-0022034521997112] Kitakawa D Oliveira FE Neves de Castro P Carvalho LFCS. 2020. Short report—Herpes simplex lesion in the lip semimucosa in a COVID-19 patient. Eur Rev Med Pharmacol Sci. 24(17):9151–9153. 32965008 10.26355/eurrev_202009_22863

[bibr19-0022034521997112] Martín Carreras-Presas C Amaro Sánchez J López-Sánchez AF Jané-Salas E Somacarrera Pérez ML. 2020. Oral vesiculobullous lesions associated with SARS-CoV-2 infection. Oral Dis [epub ahead of print 5 May 2020]. doi:10.1111/odi.13382 PMC7267423 32369674

[bibr20-0022034521997112] Morris G Bortolasci CC Puri BK Olive L Marx W O’Neil A Athan E Carvalho AF Maes M Walder K, et al. 2020. The pathophysiology of SARS-CoV-2: a suggested model and therapeutic approach. Life Sci. 258:118166. 10.1016/j.lfs.2020.118166 PMC7392886 32739471

[bibr21-0022034521997112] Nayyar SS Thiagarajan S Malik A D’Cruz A Chaukar D Patil P Alahari AD Lashkar SG Prabhash K. 2020. Head and neck squamous cell carcinoma in HIV, HBV and HCV seropositive patients—prognosis and its predictors.J Cancer Res Ther. 16(3):619–623. 32719277 10.4103/jcrt.JCRT_166_19

[bibr22-0022034521997112] Otter JA Donskey C Yezli S Douthwaite S Goldenberg SD Weber D J. 2016. Transmission of SARS and MERS coronaviruses and influenza virus in healthcare settings: the possible role of dry surface contamination. J Hosp Infect. 92(3):235–250. 26597631 10.1016/j.jhin.2015.08.027 PMC7114921

[bibr23-0022034521997112] Ottria L Lauritano D Oberti L Candotto V Cura F Tagliabue A Tettamanti L. 2018. Prevalence of HIV-related oral manifestations and their association with HAART and CD4+ T cell count: a review. J Biol Regul Homeost Agents. 32(2 Suppl 1):51–59. 29460518

[bibr24-0022034521997112] Parisi MR Tecco S Gastaldi G Polizzi E D’Amicantonio T Negri S Gardini I Schlusnus K Gherlone E Capparè P, et al. 2017. Point-of-care testing for hepatitis C virus infection at alternative and high-risk sites: an Italian pilot study in a dental clinic. New Microbiol. 40(4):242–245. 28825443

[bibr25-0022034521997112] Pascolo L Zupin L Melato M Tricarico PM Crovella S. 2020. TMPRSS2 and ACE2 coexpression in SARS-CoV-2 salivary glands infection. J Dent Res. 99(10):1120–1121. 32479133 10.1177/0022034520933589

[bibr26-0022034521997112] Peng X Xu X Li Y Cheng L Zhou X Ren B. 2020. Transmission routes of 2019-nCoV and controls in dental practice. Int J Oral Sci. 12(1):9. 32127517 10.1038/s41368-020-0075-9 PMC7054527

[bibr27-0022034521997112] Pisanic N Randad PR Kruczynski K Manabe YC Thomas DL Pekosz A Klein SL Betenbaugh MJ Clarke WA Laeyendecker O, et al. 2020. COVID-19 serology at population scale: SARS-CoV-2-specific antibody responses in saliva. J Clin Microbiol. 59(1):e02204–e02220. 10.1128/JCM.02204-20 PMC7771435 33067270

[bibr28-0022034521997112] Rovere-Querini P Tresoldi C Conte C Ruggeri A Ghezzi S De Lorenzo R Di Filippo L Farina N Ramirez GA Ripa M, et al.; COVID-BioB Study Group. 2020. Biobanking for COVID-19 research. Panminerva Med [epub ahead of print 19 Oct 2020]. doi:10.23736/S0031-0808.20.04168-3 33073557

[bibr29-0022034521997112] Saita N Fukuda K Koukita Y Ichinohe T Yamashita S. 2013. Relationship between gagging severity and its management in dentistry. J Oral Rehabil. 40(2):106–111. 23231041 10.1111/joor.12014

[bibr30-0022034521997112] Sakanashi D Asai N Nakamura A Miyazaki N Kawamoto Y Ohno T Yamada A Koita I Suematsu H Hagihara M, et al. 2020. Comparative evaluation of nasopharyngeal swab and saliva specimens for the molecular detection of SARS-CoV-2 RNA in Japanese patients with COVID-19.J Infect Chemother. 27(1):126–129. 33060046 10.1016/j.jiac.2020.09.027 PMC7524660

[bibr31-0022034521997112] Scully C Dios DP. 2001. Orofacial effects of antiretroviral therapies. Oral Dis. 7(4):205–210. 11575869

[bibr32-0022034521997112] Song J Li Y Huang X Chen Z Li Y Liu C Chen Z Duan X. 2020. Systematic analysis of ACE2 and TMPRSS2 expression in salivary glands reveals underlying transmission mechanism caused by SARS-CoV-2. J Med Virol. 92(11):2556–2566. 32441816 10.1002/jmv.26045 PMC7280739

[bibr33-0022034521997112] Stoopler ET Sollecito TP Alawi F. 2018. A white patch on the tongue. JAMA Dermatol. 154(12):1475–1476. 30090921 10.1001/jamadermatol.2018.1571

[bibr34-0022034521997112] Tecco S Sciara S Pantaleo G Nota A Visone A Germani S Polizzi E Gherlone EF. 2018. The association between minor recurrent aphthous stomatitis (RAS), children’s poor oral condition, and underlying negative psychosocial habits and attitudes towards oral hygiene. BMC Pediatr. 18(1):136. 29653566 10.1186/s12887-018-1094-y PMC5897994

[bibr35-0022034521997112] To KK Tsang OT Leung WS Tam AR Wu TC Lung DC Yip CC Cai JP Chan JM Chik TS, et al. 2020. Temporal profiles of viral load in posterior oropharyngeal saliva samples and serum antibody responses during infection by SARS-CoV-2: an observational cohort study. Lancet Infect Dis. 20(5):565–574. 32213337 10.1016/S1473-3099(20)30196-1 PMC7158907

[bibr36-0022034521997112] Usami Y Hirose K Okumura M Toyosawa S Sakai T. 2020. Brief communication: immunohistochemical detection of ACE2 in human salivary gland. Oral Sci Int [epub ahead of print 28 Sep 2020]. doi:10.1002/osi2.1085 PMC7537151 33041626

[bibr37-0022034521997112] Vaira LA Salzano G Deiana G De Riu G. 2020. Anosmia and ageusia: common findings in COVID-19 patients. Laryngoscope. 130(7):1787. 10.1002/lary.28692 PMC7228304 32237238

[bibr38-0022034521997112] Wu C Chen X Cai Y Xia J Zhou X Xu S Song Y. 2020. Risk factors associated with acute respiratory distress syndrome and death in patients with coronavirus disease 2019 pneumonia in Wuhan, China. JAMA Intern Med. 180(7):934–943. 32167524 10.1001/jamainternmed.2020.0994 PMC7070509

[bibr39-0022034521997112] Xu J Li Y Gan F Du Y Yao Y. 2020. Salivary glands: potential reservoirs for COVID-19 asymptomatic infection. J Dent Res. 99(8):989. 32271653 10.1177/0022034520918518

[bibr40-0022034521997112] Yelins’ka AM Shvaykovs’ka OO Kostenko VO. 2018. Epigallocatechin-3-gallate prevents disruption of connective tissue in periodontium and salivary glands of rats during systemic inflammation. Wiad Lek. 71(4):869–873. 30099426

[bibr41-0022034521997112] Yuen KS Ye ZW Fung SY Chan CP Jin DY. 2020. SARSCoV-2 and COVID-19: the most important research questions. Cell Biosci. 10:40. 32190290 10.1186/s13578-020-00404-4 PMC7074995

[bibr42-0022034521997112] Zangrillo A Beretta L Scandroglio AM Monti G Fominskiy E Colombo S Morselli F Belletti A Silvani P Crivellari M, et al.; COVID-BioB Study Group. 2020. Characteristics, treatment, outcomes and cause of death of invasively ventilated patients with COVID-19 ARDS in Milan, Italy. Crit Care Resusc. 22(3):200–211. 32900326 10.1016/S1441-2772(23)00387-3 PMC10692521

