# Supplementary material for: Frequent and Persistent Salivary Gland Ectasia and Oral Disease After COVID-19
Source: J Dent Res. 2021 Mar 3;100(5):464–71. doi: 10.1177/0022034521997112 (PMC7930603; doi:10.1177/0022034521997112)
Supplement: sj-pdf-1-jdr-10.1177_0022034521997112 – Supplemental material for Frequent and Persistent Salivary Gland Ectasia and Oral Disease After COVID-19 [file sj-pdf-1-jdr-10.1177_0022034521997112.pdf]

Post-COVID-19 dentistry follow-up

First name:			
Last name:			
Sex:			
Age:			
Smoking status:	never smoked	former smoker	smoker
Last check-up by the dentist:	less than one year ago	more than one year ago	more than two years ago
TMJ abnormalities	not present, or present since before Covid-19	present	
Facial pain due to facial muscle weakness	not present, or present since before Covid-19	Present	
Oral ulcers	not present, or present since before Covid-19	Present	
Dry mouth	not present, or present since before Covid-19	Present	
Facial tingling	not present, or present since before Covid-19	Present	
Trigeminal neuralgia	not present, or present since before Covid-19	Present	
Altered taste	never found	had during Covid-19 and recovered at the time of the visit	had during Covid-19 and persistent at the time of the follow-up visit
Altered smell	never found	had during Covid-19 and recovered at the time of the visit	had during Covid-19 and persistent at the time of the follow-up visit
Facial asymmetry	not present, or present since before Covid-19	Present	
Lymphadenopathy	not present, or present since before Covid-19	Present	
Anomalies of lips	not present, or present since before Covid-19	Present	
Anomalies cheeks	not present, or present since before Covid-19	Present	
Anomalies salivary glands	not present, or present since before Covid-19	Present	
Anomalies hard palate	not present, or present since before Covid-19	Present	

Anomalies oropharynx	not present, or present since before Covid-19	Present
Anomalies mucous membranes	not present, or present since before Covid-19	Present
Anomalies frenula	not present, or present since before Covid-19	Present
General dental clinical note:		
